# Editorial: Technologies to address risk assessment, food safety and public health in food production chain

**DOI:** 10.3389/fmicb.2024.1413966

**Published:** 2024-05-14

**Authors:** Ivan Nastasijevic, Beatrix Stessl, Linda Boniface Oyama, Milica Glišić

**Affiliations:** ^1^Institute of Meat Hygiene and Technology, Belgrade, Serbia; ^2^Unit of Food Microbiology, Institute of Food Safety, Food Technology and Veterinary Public Health, Department of Farm Animal and Public Health in Veterinary Medicine, University of Veterinary Medicine, Vienna, Austria; ^3^Institute for Global Food Security, School of Biological Sciences, Queen's University Belfast, Belfast, United Kingdom; ^4^Department of Food Hygiene and Technology, Faculty of Veterinary Medicine, University of Belgrade, Belgrade, Serbia

**Keywords:** food safety, risk assessment, public health, food production, systems approach, technologies

## Background

Access to sufficient, safe and nutritious food is a basic human right and is referred to as food security. To ensure this right, governments should develop and support effective and efficient prevention and control strategies along the food production chain. This should be based on integrated approach, from farm-to-fork, supported by risk assessment, risk-based food safety management and risk communication to consumers on major public health hazards. The continuous and rapid development of new technologies to detect and control food safety hazards, together with the introduction of digital innovations in the food chain, mark the 4^th^ industrial revolution. The food chain is not exempted from these changes. In light of food security needs and current global challenges, such as population growth and urbanization, international livestock and food trade, climate change, biodiversity loss, decrease of arable land, food waste, and greenhouse gas emissions there is a strong need to transform agri-food systems. Such transformation should enable a more climate resilient and sustainable food value chain.

## Goal

The dynamic growth of the global food trade in the last decade and the disruption of the food chain supply during COVID-19 pandemic have revealed many shortcomings in the national food control systems, associated with food borne outbreaks, and food recalls, thus jeopardizing public health, disrupting markets, and impacting the national economies. Therefore, there is a need for a novel and transformed food systems that considers the food chain from a holistic point of view, supported by highly effective food control through the application of state-of-the-art technologies for data collection along the food chain based on digital solutions, as well as rapid and accurate detection of food borne pathogens.

## Scope

The scope of this Research Topic covered all modules of the food chain from farm to fork in relation to microbial contamination at multiple points along the food chain. The focus was on longitudinally integrated food safety assurance system supported by detection, prevention and control of microbial food borne hazards, including novel technologies used for microbial source tracking in the food chain, as well as state-of-the-art food safety interventions. The key determinants are the use of such technologies to facilitate the transformation of food systems to become more resilient, sustainable and capable to enable sufficient quantities of safe and nutritious food, as well as novel solutions for the integrated and risk-based food safety management system that will support public health protection.

## Outcome

Within this Research Topic, 6 articles have been published that contributed to an evidence-based understanding and complemented the knowledge on available and emerging approaches and technologies to holistically address risk assessment, food safety and public health along the food production chain.

Guo L. et al. elaborated on antibacterial action of slightly acidic electrolytic water (SAEW) against *Cronobacter sakazakii* being frequently associated with contamination of Powdered Infant Formula (PIF) which can endanger the lives of newborns and infants. The antibacterial effect of SAEW on *C. sakazakii* was positively correlated to the SAEW concentration and treatment time. In addition, the action of SAEW as a disinfectant on high-risk contact surfaces, such as stainless steel and rubber was assessed and results showed that approximately 10^3^ CFU/cm^2^ of *C. sakazakii* were successfully inactivated on stainless steel and rubber surfaces after a 30 mg/L SAEW treatment for 20 s.

Guo C. et al. also elaborated on characterization and mechanism of simultaneous degradation of aflatoxin B1 (AFB_1_) and zearalenone (ZEN) by 8 types of edible fungi belonging to 6 species. Among these fungi, *Agrocybe cylindracea* strain GC-Ac2 was proved to be the most efficient in the degradation of AFB_1_ and ZEN with the degradation rate of both AFB_1_ and ZEN that reached over 96%. It was observed that the removal of AFB_1_ and ZEN was primarily degraded by the culture supernatant of the respective fungus. The mechanism of degradation of these mycotoxins is speculated to be catalyzed by a complex enzyme system, including MnP and other ligninolytic enzymes and *Agrocybe cylindracea* can degrade multiple mycotoxins and produce MnP. This is a novel and significant discovery that may be efficiently used in food and feed safety interventions.

Awad et al. carried out genotypic characterization, antimicrobial susceptibility and virulence determinants of *Campylobacter jejuni* and *Campylobacter coli* isolated from pastured poultry farms. *Campylobacter* is the leading bacterial pathogen causing foodborne illnesses worldwide and pasture farming is regarded as an important source of agricultural production for small farming communities. The Q7 BAX^®^ System Real-Time PCR and multilocus sequence typing (MLST) were used for genotypic characterization of 97 *Campylobacter* isolates. Antimicrobial susceptibility of *C. jejuni* and *C. coli* isolated against selected antimicrobial agents (tetracycline, macrolides, quinolones, fluoroquinolones, aminoglycosides, ketolide, amphenicol, and lincomycin) was done using sensititre plates. The highest resistance rate of *Campylobacter* isolates was shown against tetracycline (81.4%). *Campylobacter* isolates were also tested for the presence of antimicrobial resistance-associated elements by screening for the presence of 13 genes encoding putative virulence factors by PCR. The results of this study revealed that *Campylobacter* isolates from pasture-raised poultry farms showed genetic relatedness to *Campylobacter* isolates commonly associated with humans, indicating pasture-raised broiler flocks, similar to conventionally-reared broiler flocks, as a potential vector for antibiotic-resistant and pathogenic strains of thermophilic *Campylobacter* to humans.

Hamiot et al. investigated sporulation conditions influence the surface and adhesion properties of *Bacillus subtilis* spores, having in mind that *Bacillus subtilis* group are responsible for recurrent contamination of processing lines in the food industry which can lead to food spoilage. The particular problem is related with the persistence of *B. subtilis*; it is due to the high resistance of spores to extreme environmental condition and their propensity to contaminate surfaces. The impact of 13 sporulation conditions on the surface and adhesion properties of 168 spores of *B. subtilis* was studied. It was shown that Ca^2+^ or Mg^2+^ depletion, lower oxygen availability, acidic pH, and oxidative stresses during sporulation lead to the release of more hydrophobic and adherent spores. In addition, the consequences of these sporulation conditions on crust composition in carbohydrates and proteins were also evaluated. It was observed that that lower oxygen availability or addition of hydrogen peroxide during sporulation decreases the relative amount of two crust proteins (CgeA and CotY). The authors concluded the fact that sporulation conditions affect the ease with which spores can contaminate surfaces could explain the frequent and recurrent presence of *B. subtilis* spores in food processing lines.

Nouws et al. elaborated on transforming Shiga toxin-producing (STEC) *Escherichia coli* surveillance via whole genome sequencing (WGS) in food safety practices. WGS in STEC surveillance is promising tool in outbreak prevention and confinement, in broadening STEC epidemiology and in contributing to risk assessment and source attribution. Despite international recommendations, WGS is often restricted to assist outbreak investigation and is not yet fully implemented in food safety surveillance across all European countries, in contrast to for example in the United States. WGS was retrospectively applied to isolates collected within the context of Belgian food safety surveillance and combined with data from clinical isolates to evaluate its benefits. A cross-sector WGS-based collection of 754 strains from 1998 to 2020 was analyzed. The authors confirmed that WGS in food safety surveillance allows accurate detection of genomic relationships between human cases and strains isolated from food samples, including those dispersed over time and geographical locations. This can reveal new insights into outbreaks and direct epidemiological investigations to facilitate foodborne outbreak management. This study contributed to understanding of developing a representative WGS-based collection of circulating STEC strains in food chain and within consumers and, by illustrating its benefits, it aims to encourage policymakers to support WGS uptake in the national food safety surveillance system.

De Bock et al. discussed on the importance and usage of microbiological profiling and knowledge of food preservation technology to support guidance on a neutropenic diet for immunocompromised patients, having in mind the increasing number of people vulnerable to infections. It implies to people with severe immunodeficiency, to whom a neutropenic or low-microbial diet is frequently being prescribed; such diet aims to substitutes high-risk foods, that are more likely to contain human (opportunistic) pathogens, with lower-risk alternatives. However, the neutropenic dietary guidelines are typically set up from a clinical and nutritional perspective, rather than from a food processing and food preservation perspective. In this study, the current guidelines by the Ghent University Hospital were evaluated based on the current knowledge of food processing and preservation technologies. Three criteria, used as a framework for the evaluation of the suitability of foodstuffs to be included in a low-microbial diet, were identified as important: (1) the microbial contamination level and composition; (2) the potential presence of established foodborne pathogens such as *Salmonella* spp. (a zero-tolerance policy is recommended); and (3) an increased vigilance for *Listeria monocytogenes* as an opportunistic foodborne pathogen with a high mortality rate in immunocompromised individuals (a zero-tolerance policy should apply). A restricted screening on a selection of (minimally processed) plant-based foodstuffs on the retail market in Flanders, Belgium supported decision-making on the inclusion of these food types in a low-microbial diet. The authors concluded that when determining the suitability of a foodstuff to be included in a low-microbial diet, not only the microbiological status but also nutritional and sensorial properties should be assessed, which requires multidisciplinary communication and collaboration.

## Author contributions

IN: Conceptualization, Methodology, Supervision, Validation, Writing – original draft, Writing – review & editing. BS: Validation, Writing – original draft, Writing – review & editing. LO: Validation, Writing – original draft. MG: Validation, Writing – original draft, Writing – review & editing.

